# Patient-Reported Outcomes After Periodontal Surgical Procedures

**DOI:** 10.7759/cureus.63818

**Published:** 2024-07-04

**Authors:** Devashri P Newaskar, Prathamesh N Motadu

**Affiliations:** 1 Periodontology, Bharati Vidyapeeth Deemed to be University Dental College and Hospital, Pune, IND; 2 Prosthodontics, Sinhgad Dental College and Hospital, Pune, IND

**Keywords:** patient-reported outcome measures, soft tissue grafting procedures, periodontal surgeries, open flap debridement, crown lengthening

## Abstract

Background

Patient satisfaction is the primary focus of the healthcare system. Patient-reported outcome measures (PROMs) are standardized and valid measures obtained directly from the patients and are used to assess and compare the efficacy of healthcare services. This can help improve the service provided by the healthcare system. Therefore, this study aimed to assess PROMs during the first week post-surgery across different periodontal surgical procedures and explore their correlation with surgical duration. Furthermore, the study sought to evaluate the occurrence of postoperative complications.

Methodology

A total of 30 healthy patients with no systemic history, requiring periodontal surgical procedures such as crown lengthening (CLP), open flap debridement (OFD), and soft tissue grafting (STG) were included in the study. The Visual Analog Scale (VAS) was utilized for gathering PROMs concerning bleeding, swelling, bruising, and pain at intervals of days zero, three, five, and seven after the surgical procedure.

Results

On the surgical day and over seven days, VAS scores were the lowest for CLP and highest for STG procedures. This result is in accordance with the duration required for surgery. VAS scores for OFD were intermediate. Prevalence of 20% soft tissue graft dehiscence and 40% tenderness on palpation was observed. Swelling and bleeding were noticed in 10% and 20% of OFD cases.

Conclusions

One week post-surgically, the mean VAS scores were minimum for CLP, whereas maximum for STG procedures. As CLP and OFD require less duration compared to STG, duration plays a significant role in post-surgical outcomes. Prevalence of the post-surgical complications is also related to the duration of the surgery.

## Introduction

Patient-reported outcomes (PROs) have gained significant attention in the assessment of healthcare services. These outcomes are valuable because they provide insights directly from patients about their health conditions, quality of life, and the effectiveness of treatments. Healthcare providers and systems use PROs to measure the quality and performance of their services. By understanding patients’ perspectives on their health outcomes, providers can identify areas needing improvement and make data-driven decisions to enhance care quality. They strive to deliver more effective service for the best interest of patients. A vital aspect of patient-centered outcomes research and comparative effectiveness research entails integrating patients’ perspectives on their health with clinical and biological data to evaluate the efficacy and safety of interventions. This integration recognizes the influence of disease and treatment on health-related quality of life, enhancing traditional clinical endpoints [1]. PROs are directly furnished by the patient without interpretation by a clinician or other parties, concentrating on dimensions such as health, quality of life, or functional status of healthcare or treatment, and are evaluated using patient-reported outcome measures (PROMs). PROMs consist of standardized, validated questionnaires completed by patients to assess their perceptions of functional status and well-being [2,3]. This definition underscores the outcomes and distinguishes them from questionnaires aimed at evaluating patients’ experiences during the care process. They assess the quality of service provided to patients. Periodontal surgical treatment is now routine in current clinical practice, and there is a demand to get some type of standard information on how the surgeries have turned out for patients. While assessment has always occurred, it is being defined more clinically now. PROM serves as a tool for this assessment and to transfer the knowledge to other people which makes it revolutionary. Including PROMs to document patients’ experiences of bleeding, swelling, pain, and bruising during the first week of postoperative recovery can be pivotal for managing patient expectations. The Visual Analog Scale (VAS) serves as a tool for estimating patients’ perceptions. The validity and reliability of VAS have been proven by many studies [4-7]. The VAS stands out as one of the most effective methods for estimating the study’s outcome variables, as it provides an objective quantification of subjective perceptions. Tan et al. in 2014 [8] used VAS to compare PROMs after periodontal and implant surgical procedures among dentate subjects.

Despite being a significant and relevant clinical aspect of treatment, PROMs after surgical procedures are often underreported. McGrath et al. in 2012 [9] reported a review of PROMs after dental implant surgical procedures. However, the limited literature in this area of periodontics raises a need to evaluate post-surgical patient experience and assess post-surgical sequelae of different periodontal surgical procedures. Thus, PROs are a vital component of modern healthcare, providing valuable insights into patient experiences and outcomes, ultimately leading to more effective and patient-centered care.

Hence, a study was designed to compare VAS scores for all PROM parameters (bleeding, swelling, pain, and bruising) over the week following periodontal surgical procedures, while also examining the correlation between VAS scores and the duration of the surgical procedure.

The objective of the current study was to quantify PROMs for various types of periodontal surgeries during the initial post-surgical week and assess the prevalence of post-surgical complications based on the duration of surgery.

## Materials and methods

Eligibility criteria

The study received ethical approval from Yashwantrao Chavan Memorial Medical & Rural Development Foundation’s Dental College and Hospital (approval number: YCMM&RDF's/IERB/10) All subjects enrolled for the study were systemically healthy in the age range of 20 years to 45 years. Selected subjects were indicated for crown lengthening (CLP) surgery, flap surgery, and soft tissue grafting (STG) procedures which included either free gingival graft or connective tissue graft procedure. Subjects who had a history of any systemic disease and were on medications were excluded from the study as it could interfere with the results obtained. Patients with adverse habits and pregnant or lactating mothers were not included in this study.

Sample size estimation

The sample size was determined using the estimates of mean and standard deviation values from Tan et al.(2014) [8] using the following formula: n = (Z1-α/2+ Z1-β)^2^ [s]^2 ^/d2, where Z1-α/2 is the z variate of alpha error, i.e., a constant with a value of 1.96, Z1-β is a constant with a value of 0.84. Approximate estimates were 80% power, type I error of 5%, type II error of 20%, and a true difference of at least 0.52 units between the time intervals. The pooled standard deviation was 0.99. Substituting the values, n = (2.8)^2^ [0.99]^2^/ (0.52)^2 ^28.42. Hence, approximately 28 subjects were needed for the study per time interval until the endpoint. According to statistics, to apply a parametric test and for the normality of data and considering the attrition of samples, the minimum sample size estimated was 30. Thus, the minimum sample size required in each group was 10 with an alpha error of 0.01.

Study population

Patients referred to the Department of Periodontics of our college from 2016 to 2017 were screened and 30 patients indicated for various periodontal surgical procedures such as CLP, open flap debridement (OFD), and STG were enrolled for the study. Patients were informed that the participation was voluntary. Relevant informed consent was obtained by respective surgeons. Patients were evenly divided into three groups, depending on the periodontal surgical procedure required.

Surgical procedures

The study participants were informed about the details of the procedure of their respective surgeries. Patient counseling was done to reduce the anxiety level. Research has demonstrated that each of the three domains of psychoeducational content comprising healthcare-relevant information, skills teaching, and psychological support when delivered individually has been linked to positive effects [10].

Patients underwent their scheduled surgeries. Sterile instruments and aseptic techniques were utilized for all surgeries, accompanied by the administration of an adequate amount of anesthesia at the operation site.

Depending on the type of procedure required, the following surgeries were performed: Group A: CLP surgery with osseous resection; Group B: Periodontal flap surgery (Kirkland flap surgery, modified Widman flap surgery); and Group C: STG procedures such as free gingival graft surgery and subepithelial connective tissue graft surgery to augment the attached gingiva width or root coverage.

**Figure 1:**
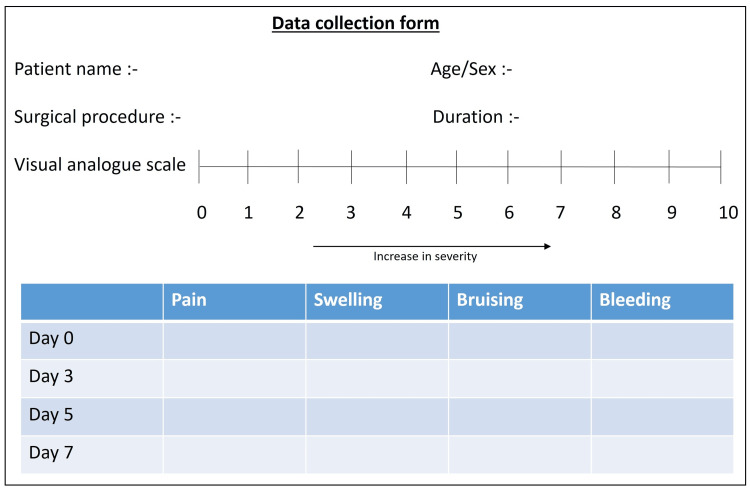
Data collection form.

After completion of the procedure, patient perceptions on the day of surgery followed by the third, fifth, and seventh day and the duration of the surgery were charted on the data collection form (Figure 1) with the VAS charting system designed for this study. A score of 0 indicated an absence of pain/bleeding/swelling/bruising and 10 designated severe pain/bleeding/swelling/bruising. The data collection form used for the study, mentioning patient details, the type of procedure undergone, and scores obtained on VAS is shown in Figure 1.

Patients were instructed regarding charting their perceptions of the primary outcome variables such as pain, bleeding, swelling, and bruising over the first-week healing period, i.e., the third, fifth, and seventh days using the VAS provided. On the seventh day when patients returned for suture removal, the presence of any post-surgical complication was also assessed. The anonymity and confidentiality of the data obtained from the participants was maintained.

Outcome variables

Primary outcome variables were PROMs assessed through VAS scores on pain, bleeding, swelling, and bruising. The secondary outcome variable was post-surgical complications such as flap dehiscence, tenderness on palpation, and suppuration on day seven.

Statistical analysis

Data were compiled in an MS Office Excel worksheet (Microsoft Corp., Redmond, WA, USA) and subjected to statistical analysis using SPSS software (IBM Corp., Armonk, NY, USA). Descriptive statistics such as frequency (n) and percentage (%) of categorical data and mean and standard deviation of numerical data in each time interval were depicted. The normality of numerical data was checked using the Shapiro-Wilk test or Kolmogorov-Smirnov test. Depending on the normality of the data, statistical tests were determined. Intragroup comparisons for numerical continuous data following a normal distribution were done using the paired t-test (for two observations) and repeated measures were done by analysis of variance for more than two observations. Frequency (n) and percentage (%) of various categories in each time interval were compared using the chi-square test. With an alpha error of 5%, a beta error of 20%, and a power of 80%, p-values <0.05 were considered statistically significant.

## Results

Demographics

Table 1 presents the details of the study participants according to the surgical procedure. In total, 30 healthy patients needing periodontal surgeries were recruited, with 13 (43%) males and 17 (57%) females. Surgeries performed were CLP surgery with osseous resection, OFD, and STG procedures such as free gingival graft procedure and subepithelial connective tissue graft procedures with 10 subjects each. Overall, for 57% (n = 17) of surgeries, the duration was more than 60 minutes.

**Table 1:** Patient demographics according to surgical procedures. CLP = crown lengthening procedure; OFD = Open flap debridement; STG = soft tissue graft procedures

Variable		All surgeries	Surgical procedure type		
		Number of subjects (N = 30)	CLP	OFD	STG
Gender	Males	13 (43.33%)	2 (20%)	4 (40%)	6 (60%)
	Females	17 (56.66%)	8 (80%)	6 (60%)	4 (40%)

Surgical procedures

Table 2 presents the comparison of mean VAS scores for all procedures on the day of surgery (day zero) which reduced significantly over day seven. The reduction of VAS scores of primary outcome measures was slowest for STG procedures. The mean VAS scores were the lowest for CLP, intermediate for OFD, and highest for STG procedures. Analysis of variance (p < 0.01) showed that the variance reached statistical significance. Similarly, a statistically significant difference (p < 0.01) was also observed for the score of all procedures on the day of surgery and the seventh-day post-surgery. Bleeding was found to be significantly less compared to pain. Post-surgical complications included 20% STG dehiscence and 40% tenderness on palpation. Swelling and bleeding were observed in 10% and 20% of OFD cases, respectively.

**Table 2:** Comparison of the mean VAS scores. *: statistically significant (p < 0.01). VAS = Visual Analog Scale; PROM = patient-reported outcome measure; CLP = crown lengthening procedure; OFD = Open flap debridement; STG = soft tissue graft procedures

PROM parameter	Day	CLP, mean (SD)	OFD, mean (SD)	STG, mean (SD)
Pain	0	2.5 (0.0)	5.9 (0.57)	7.1 (0.99)*
	3	1.5 (0.53)	3.7 (0.64)	5.2 (0.63)
	5	2.5 (0.0)	0.2 (0.42)	2.8 (0.91)
	7	0 (0.0)*	0.0 (0.0)*	1.9 (0.87)*
Swelling	0	2.1 (0.32)	7.0 (1.50)	5.6 (0.69)*
	3	0.8 (0.42)	4.9 (1.66)	4.0 (0.94)
	5	0.0 (0.0)	3.0 (1.41)	2.6 (0.96)
	7	0.0 (0.0)*	0.1 (0.32)*	1.7 (0.67)*
Bruising	0	1.4 (0.69)	0.9 (1.52)	6.6 (1.07)*
	3	0.3 (0.48)	0.3 (0.67)	6.1 (1.59)
	5	0.0 (0.0)	0.2 (0.42)	4.6 (1.95)
	7	0.0 (0.0)*	0.0 (0.0)*	2.8 (1.47)*
Bleeding	0	0.7 (0.67)	0.7 (0.95)	6.3 (0.94)*
	3	0.0 (0.0)	0.4 (0.69)	3.8 (1.13)
	5	0.0 (0.0)	0.3 (0.48)	2.4 (1.17)
	7	0.0 (0.0)*	0.2 (0.42)*	0.0 (0.69)*

Duration of surgeries

**Figure 2:**
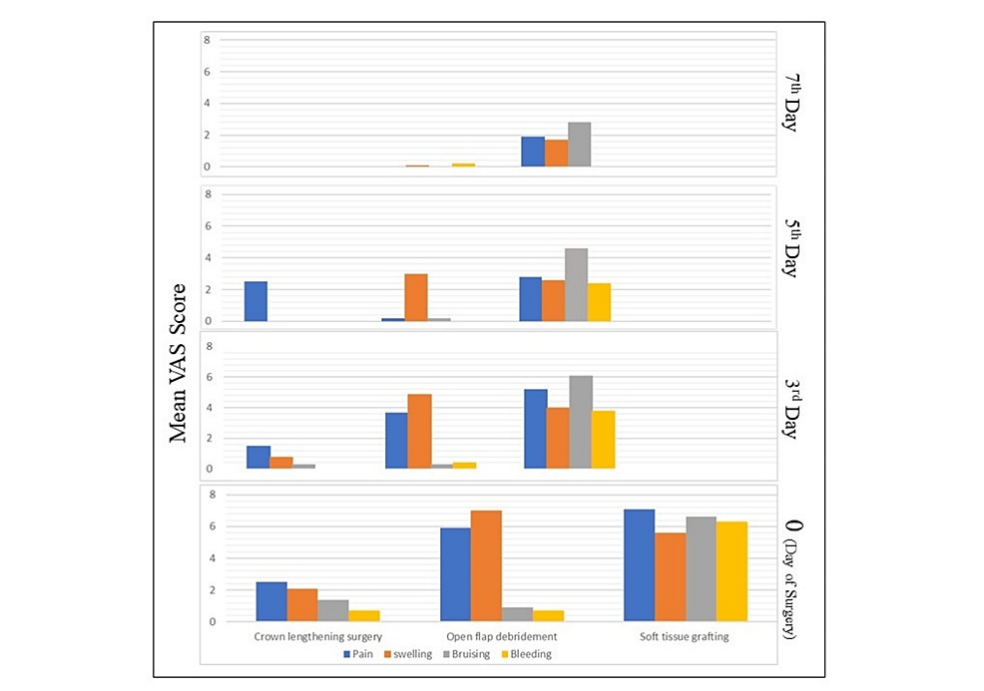
Mean VAS scores of pain, swelling, bruising, and bleeding (PROM parameters) for periodontal surgical procedures on the day of surgery (day zero), day three, day five, and day seven. PROM = patient-reported outcome measures; VAS = Visual Analog Scale

Figure 2 shows that the mean VAS scores were the highest on the day of surgery (day zero) and reduced significantly by day seven. The VAS scores were the highest for STG procedures compared to CLP and OFD. The mean VAS score for CLP and OFD procedures was reduced to almost 0 by the seventh day except for STG procedures. The mean VAS scores for CLP were significantly lower than those for STG procedures.

Table 3 shows out of 30 periodontal surgical procedures, 13 surgeries took <60 minutes, and the mean VAS score for those surgeries was significantly lower than surgeries of longer duration, i.e., >60 minutes. This proves that the duration of surgery significantly affects post-surgical outcomes.

**Table 3:** VAS scores according to duration. VAS = Visual Analog Scale

Time required	Number of surgeries	VAS scores, mean ± SD
<60 minutes	13	0.46 ± 0.826
>60 minutes	17	2.87 ± 2.592
Total	30	1.94 ± 2.400

## Discussion

Surgical procedures are followed by a variable amount of morbidity which includes pain, swelling, bleeding, and bruising, which is a subjective perception. These issues are also attributed to periodontal surgeries. A well-designed objective scale measures these patient responses to quantify the post-surgical outcomes. A VAS-based questionnaire can appropriately grade these responses and differentiate outcomes between two procedures that are significantly different clinically. This will aid in understanding the outcomes of surgery without a subjective bias. These quantitative values will help improve the quality of healthcare provided.

The current VAS-based questionnaire study revealed that patients’ perceptions of bleeding, swelling, pain, and bruising during the initial week of post-surgical healing were generally modest, with most cases being evaluated as well tolerated, as indicated by a reduction to a score of 0 on day seven for the majority of patients. The study identified significant differences in PROMs between CLPs, flap surgeries, and STG procedures (p < 0.01). The mean VAS scores for all PRO parameters in CLP were the lowest and were the highest with STG procedures.

Wessel and Tatakis conducted a study (2008) [11] comparing patient outcomes following subepithelial connective tissue graft (CTG) and free gingival graft (FGG) procedures and reported that FGG is linked to a higher incidence of donor site pain compared to CTG during the early postoperative period. Therefore, STG procedures are associated with greater analgesic usage. A study on patients’ perception of postoperative pain after periodontal flap surgery by Canakçi and Canakçi (2007) [12] reported that there were no statistically significant differences between surgical procedures of modified Widman flap, flap with osseous resection, or gingivectomy but postoperative pain experienced was greater in flap with osseous resection and gingivectomy than scaling and root planning and modified Widman flap. Tonneti et al. (2004) [13] conducted a separate investigation on patients’ perspectives of outcomes after regenerative therapy for deep intrabony defects which revealed minimal intraoperative or postoperative pain or discomfort associated with papilla preservation flap surgery, regardless of Emdogain application. The study emphasized early healing events, pain, discomfort, and adverse events linked with the surgery along with patient perceptions regarding the advantages and disadvantages of periodontal surgery in intrabony defects over one year. Although Emdogain demonstrated quicker improvements in soft tissue density, both techniques displayed comparable trends in terms of patient-centered outcomes. In a study by Hashem et al. (2006) [14], pain and anxiety levels post-implant surgeries were scrutinized, revealing an average pain score of 24/100 on day one post-surgery. Likewise, Al-Khabaaz et al. (2007) [15] found that the highest mean pain scores were documented on the day of the surgery.

Rokn et al. (2020) [16] conducted a randomized control trial and compared the use of collagen mucograft versus FGG to augment keratinized tissue width around Teeth. The study concluded that collagen mucograft can be used as an alternative to FGGs as it reduced the surgical time and patients experienced less pain.

Fu et al. (2021) [17] compared PROMs and clinical outcomes after augmentation with xenogeneic collagen matrix (XCM) or FGG during different postoperative phases and concluded that the bleeding and swelling related to the surgery were considerable during the first three postoperative days. Compared with XCM, FGG caused non-significantly greater bleeding and swelling, which is inconsistent with previous findings. The study also suggested that proper surgical procedures could result in similar bleeding and swelling levels for FGG and XCM procedures.

In this study, the duration of the surgical procedure was a significant factor influencing PROMs during the first week of healing. Procedures lasting 60 minutes or more were statistically linked to higher VAS scores for swelling and bruising on the day of surgery compared to those lasting less than 60 minutes. Moreover, at the one-week post-surgery mark, patients who underwent procedures lasting 60 minutes or longer exhibited significantly higher VAS scores (2.87 ± 2.592) for swelling, pain, and bruising in comparison to those who underwent procedures lasting under 60 minutes (0.46 ± 0.826).

The findings of this study also align with a study by Griffin et al. (2006) [18] who reported an increase in post-surgical complications with longer surgery durations, highlighting the strong association between surgical duration and pain and swelling following gingival augmentation procedures.

This study has a few limitations. Owing to patient-reported data, the data are entirely subjective, and patient perception may vary according to individuals. Other parameters such as changes in esthetics, root hypersensitivity, and chewing efficacy which contribute to changes in oral health-related quality of life were not evaluated. Operator variability may also alter the tissue response. Oral hygiene maintenance followed by the patients at home could not be monitored. Hence, further studies should be conducted to eliminate the drawbacks of the study which will include reducing the operator variability and evaluating PROMs of medically compromised patients and patients with parafunctional habits which significantly contribute to wound healing and the outcomes of surgical procedures.

## Conclusions

The mean VAS scores for all PROM parameters (pain, swelling, bleeding, and bruising) approached nearly zero on the seventh day after periodontal surgeries. Overall, the mean VAS scores for the parameters were the lowest for CLP and highest for STG procedures, while remaining moderate for OFD, which correlates with the duration required for surgeries. Thus, shorter surgery durations correlated with lower VAS scores across all PROM parameters. Considering PROs is valuable to improve the oral health-related quality of life by evaluating postoperative symptoms, making it a crucial part of clinical practice.
